# Supplementary material for: Comprehensive Volatilome and Metabolome Signatures of Colorectal Cancer in Urine: A Systematic Review and Meta-Analysis
Source: Cancers (Basel). 2021 May 21;13(11):2534. doi: 10.3390/cancers13112534 (PMC8196698; doi:10.3390/cancers13112534)
Supplement: Supplementary file 1 [file cancers-13-02534-s001.zip › proofed supp/SR-MA_Urine_CRC_VOL-MET_SuppFigures_vSubmitted_corrected.docx]

Comprehensive volatilome and metabolome signatures of colorectal cancer in urine: A systematic review and meta-analysis

Celia Mallafré-Muro^1,2, ɫ^, Maria Llambrich^3,ɫ^, Raquel Cumeras^3,4,5^, Antonio Pardo^1^, Jesús Brezmes^3,4^, Santiago Marco^1,2^, Josep Gumà^6^.

Supplementary Figures


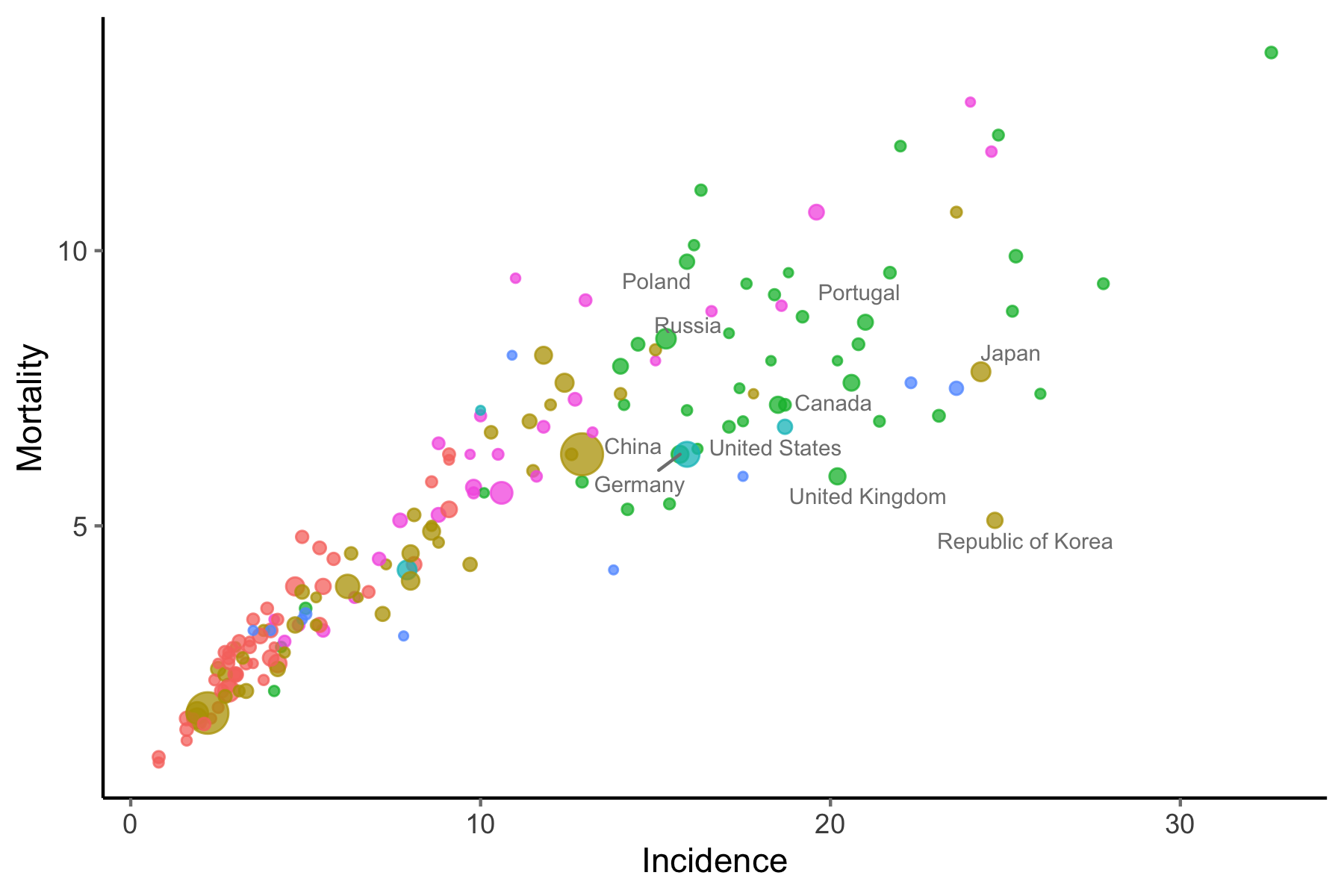


*Figure S1: Mortality versus incidence data from countries worldwide, with names of the countries of the studies from which this review is based on. The image is based on data from [42]. The circle size is proportional to the country’s population. The color indicates the continent: pink Africa, brown Asia, green Europe, light blue North America, navy Australia, purple South America and Central America.*


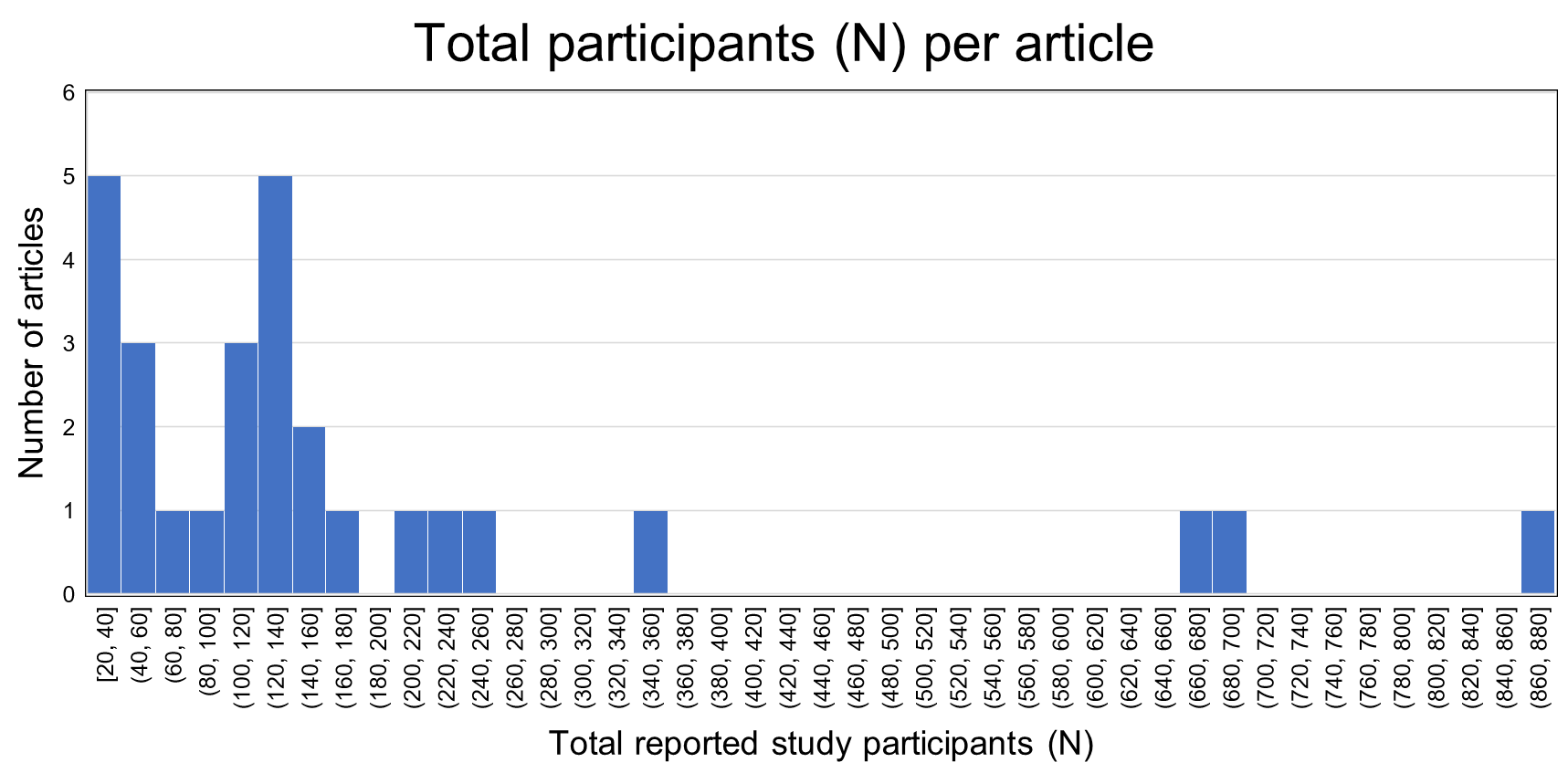


*Figure S2: Histogram of the total reported number of participants (N) per study included in the articles selected for the systematic review.*


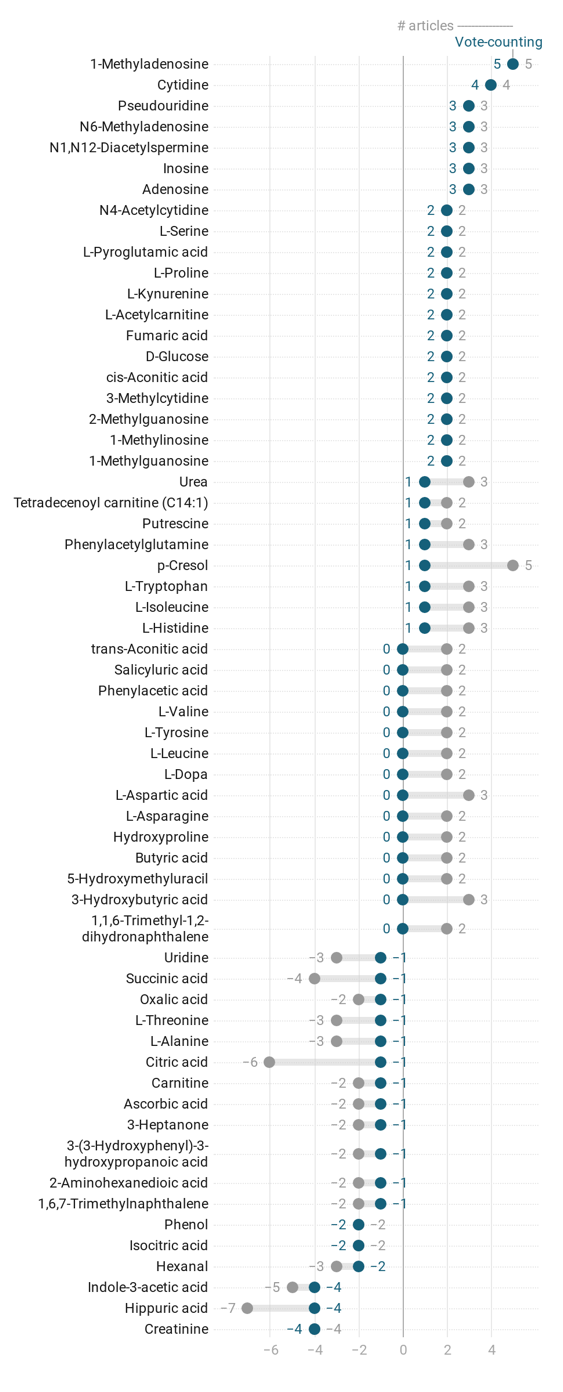


Figure S3: Qualitative vote-counting of colorectal cancer-related compounds as a range plot between the vote-counting values (blue) and the total number of articles (gray) from which the vote-counting is calculated. Positive values are compounds upregulated in CRC, while negative values are compounds downregulated in CRC.


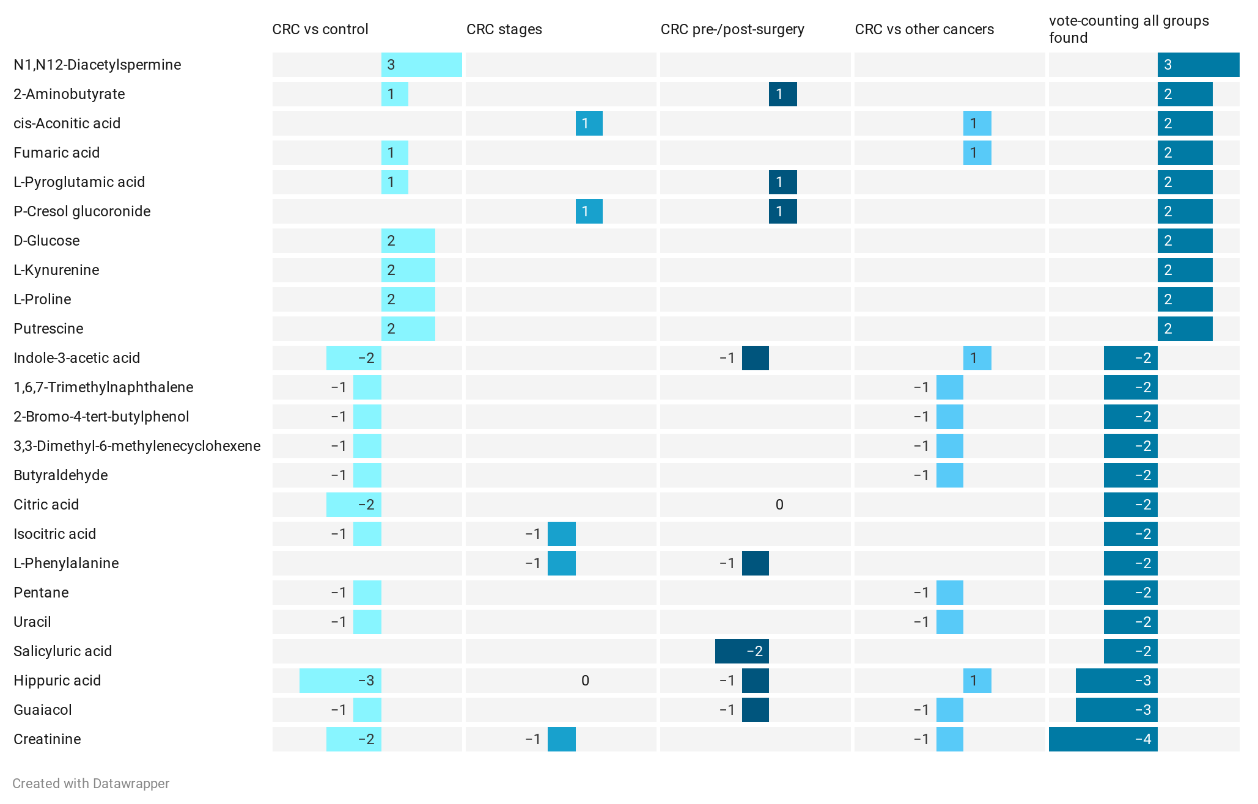


*Figure S4: Vote-counting plot for the meta-analysis results per group.*


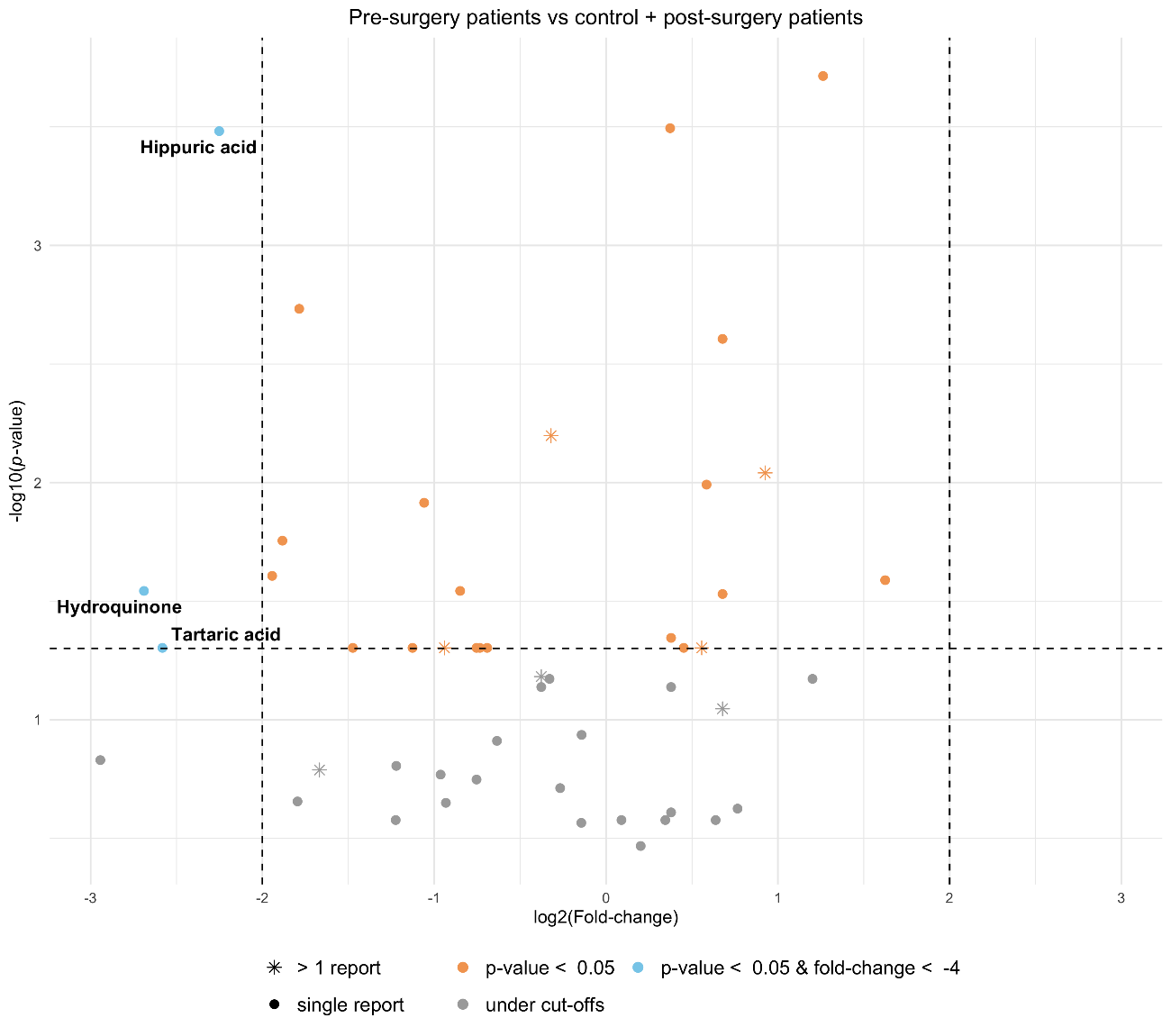


*Figure S5: Volcano plot of compounds with consistent classification from the meta-analysis comparing colorectal cancer pre-surgery vs. post-surgery. ⁕ Indicates the compound was found in more than 1 study.*


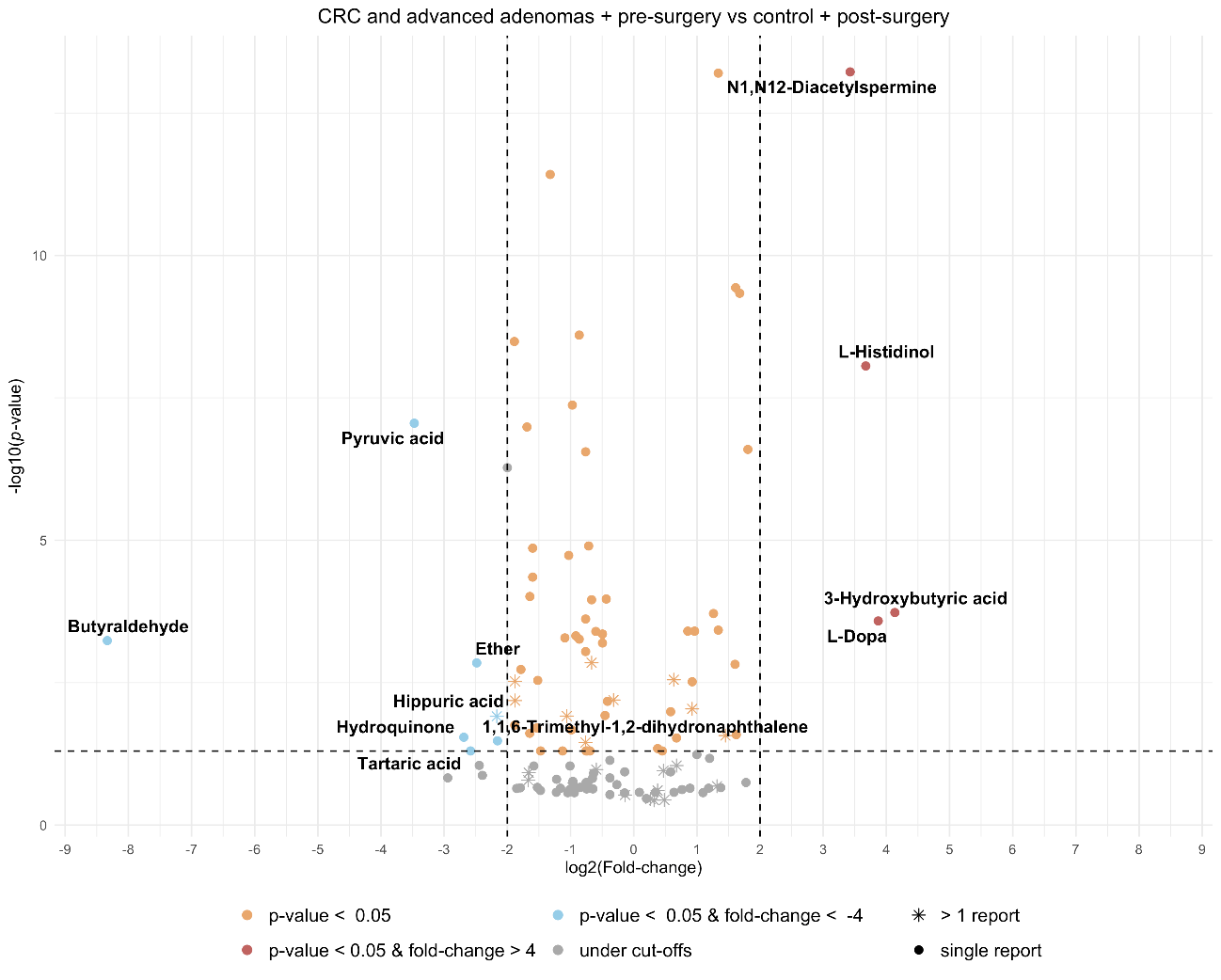


*Figure S6: Volcano plot of compounds with consistent classification from the meta-analysis comparing colorectal cancer and advanced adenomas + pre-surgery vs. control + post-surgery. ⁕ Indicates the compound was found in more than 1 study.*
